# Role of Two Exceptional *trans* Adenylation Domains and MbtH‐like Proteins in the Biosynthesis of the Nonribosomal Peptide WS9324A from *Streptomyces calvus* ATCC 13382

**DOI:** 10.1002/cbic.202000142

**Published:** 2020-06-18

**Authors:** Mirjam Bernhardt, Stefanie Berman, David Zechel, Andreas Bechthold

**Affiliations:** ^1^ Department of Pharmaceutical Biology and Biotechnology University of Freiburg Stefan-Meier-Strass 19 79104 Freiburg im Breisgau Germany; ^2^ Department of Chemistry Queen's University 90 Bader Lane Kingston, Ontario K7 L 3 N6 Canada

**Keywords:** adenylation domains, biosynthesis, mutagenesis, nonribosomal peptide synthetases, WS9326A

## Abstract

Nonribosomal peptide synthetases (NRPS) are organized in a modular arrangement. Usually, the modular order corresponds to the assembly of the amino acids in the respective peptide, following the collinearity rule. The WS9326A biosynthetic gene cluster from *Streptomyces calvus* shows deviations from this rule. Most interesting is the presence of two *trans* adenylation domains that are located downstream of the modular NRPS arrangement. Adenylation domains are responsible for the activation of their respective amino acids. In this study, we confirmed the involvement of the *trans* adenylation domains in WS9326A biosynthesis by performing gene knockout experiments and by observing the selective adenylation of their predicted amino acid substrates *in vitro*. We conclude that the *trans* adenylation domains are essential for WS9326A biosynthesis. Moreover, both adenylation domains are observed to have MbtH‐like protein dependency. Overall, we conclude that the *trans* adenylation domains are essential for WS9326A biosynthesis.

## Graphical abstract

**Rule breakers**: The *trans*‐acting adenylation domains Cal22_Thr_ and Cal23_Asn_ are shown through gene disruption experiments and *in vitro* assays with purified enzymes to play essential roles in the biosynthesis of the nonribosomal peptide WS92326A in *S. calvus*. For soluble heterologous protein production, Cal22_Thr_ and Cal23_Asn_ require coexpression with the MbtH‐like protein Cal4, the latter copurifying with Cal23_Asn_ as a complex.

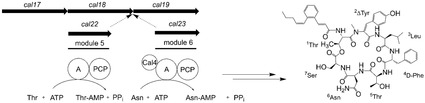

## Introduction

Nonribosomal peptides (NRPs) represent one of the most prominent classes of microbial secondary metabolites.^1^ These compounds appear as vital drugs in pharmaceutical therapy, due to their ability to exhibit antibiotic activities, like vancomycin^2^, ^3^ and daptomycin^4^ or immunosuppressor activities like cyclosporine A.^5^ NRPs are synthesized by large multienzyme complexes, called nonribosomal peptide synthetases (NRPSs). A NRPS consists of multiple modules, the number of which usually corresponds to the number of the amino acids incorporated in the NRP. Each module is required to add one distinct amino acid to the growing NRP.^1^ To perform this task, each module consists of several domains, some of them with essential functions and others with optional modifying functions. Essential domains are adenylation (A) domains, peptidyl carrier protein (PCP) domains and condensation (C) domains. Incorporation of each amino acid during NRP biosynthesis starts with the activation of the corresponding amino acid. A‐domains catalyze this step in an adenosine triphosphate (ATP) dependent fashion by selecting and adenylating the required amino acid with concomitant release of pyrophosphate (PP_i_). The amino acid substrate is recognized within the A‐domain active site by ten first‐shell residues. Two of these residues are conserved, while eight variable amino acid residues, along with second shell residues, enable recognition of the substrate amino acid side chain, thereby dictating the substrate specificity of the A‐domain.^1^ The specificity of A‐domains can therefore be predicted from the active site sequence motif by NRPSpredictor2^6^ and Stachelhaus code.^7^ This is known as the nonribosomal code.^1^

In a second step of NRP biosynthesis, the A‐domain condenses the amino acyl adenylate with the thiol terminus of the phosphopantetheinyl cofactor of the PCP‐domain. Subsequently, the C‐domain catalyzes peptide bond formation through the addition of the α‐amino group of a downstream PCP bound amino acid to the thioester of the upstream PCP bound amino acid or peptide chain. Synthesis of the peptide chain is completed when the peptide chain reaches the final module, whereupon a thioesterase domain (TE) catalyzes either macrocyclization or hydrolysis of the chain to release the final product.^1^

Genes encoding for MbtH‐like proteins (MLPs) are found in NRPS gene clusters.^10^ These small proteins are homologous to the MbtH protein encoded within the mycobactin gene cluster of *Mycobacterium tuberculosis*.^11^ Boll et al. observed that A‐domain activity is often dependent upon MLPs and that adenylation activity is observed to increase *in vitro* in the presence of MLPs.^12^ The MLP YbdZ from the *Escherichia coli* enterobactin biosynthetic pathway can interact with recombinant A‐domains and therefore affect their true biochemical properties in enzymatic assays. To avoid this problem, Boll et al. used a *ybdZ* deficient *E. coli* strain for heterologous A‐domain production. Unfortunately, the *ybdZ* knockout led to a reduction in soluble protein yields.^12^ An allosteric effect of MLP's on A‐domains has been confirmed.^13^, ^14^ Based on these observations, MLPs are postulated to enhance A‐domain solubility and adenylation activity.^10^, ^12^

The number and order of the amino acids in the NRP typically corresponds to the number and order of the individual modules of the NRPS, a relationship called collinearity.^1^, ^15^ There are exceptions from this modular arrangement and some modules are used iteratively or in a different order than their gene cluster sequence.^1^ Such deviations from collinearity can add versatility to NRP biosynthesis.^15^ A nonlinear NRPS is found in viomycin biosynthesis, in which a *trans* working A‐domain is proposed to catalyze the amino acid adenylation in a module, lacking an A‐domain.^16^ Other examples for *trans* working A‐domains are found in the respective NRPSs for andrimid,^17^ syringomycin^18^ and yersiniabactin.^19^ Andrimid is remarkable for its highly dissociated NRPS, where every A‐domain is free‐standing.^17^ In yersiniabactin biosynthesis, the A‐domain HWMP2 is responsible for the incorporation of three cysteine residues. One cysteine is incorporated in *cis* to the corresponding PCP domain, while the other two are incorporated in *trans* to the PCP domains of modules 3 and 5.^19^ During syringomycin biosynthesis, the first A domain may work in *trans* with the ninth module to incorporate a threonine residue.^15^, ^18^ These unusual mechanisms are not often found in NRPS and are therefore of great interest.

**Figure 1:**
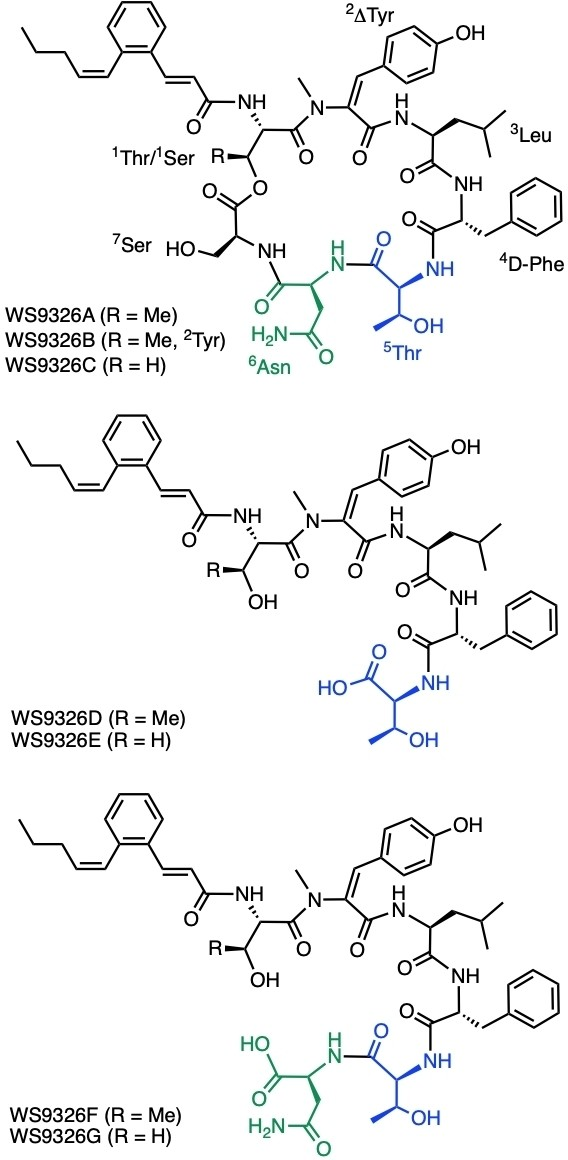
Structures of WS9326A and related congeners. Amino acids introduced by the *trans* acting A domains Cal22_Thr_ and Cal23_Asn_ are colored blue and green, respectively.^8^, ^9^

*Streptomyces calvus* ATCC 13382 synthesizes the NRP WS9326A.^20^ This NRP is a cyclic depsipeptide formed from seven amino acids (Figure 1). Additionally, a (*Z*)‐pentenylcinnamoyl side chain is linked to the first amino acid.^21^ This polyketide moiety is also found in other NRPs, such as skyllamycins,^22^, ^23^ pepticinnamins^24^ and in mohangamide A and B.^25^ WS9326A was first isolated from *Streptomyces violaceusniger* sp. 9326 as a tachykinin receptor antagonist.^26^ Subsequently, Yu and co‐workers identified WS9326A and the congeners C, D and E in extracts of *Streptomyces* sp. 9078.^9^ Recently, *S. asterosporus* DSM 41452 was shown to produce WS9326A, B, D, E, F and G.^8^ Notably, WS9326D and E are truncated congeners of WS9326A, missing asparagine 6 (^6^Asn) and serine 7 (^7^Ser; Figure 1). WS9326F and G are also truncated congeners, but in this case only missing ^7^Ser. The WS9326A gene cluster has been identified in the genomes of *S. asterosporus* 8 and *S. calvus*.^21^

**Figure 2:**
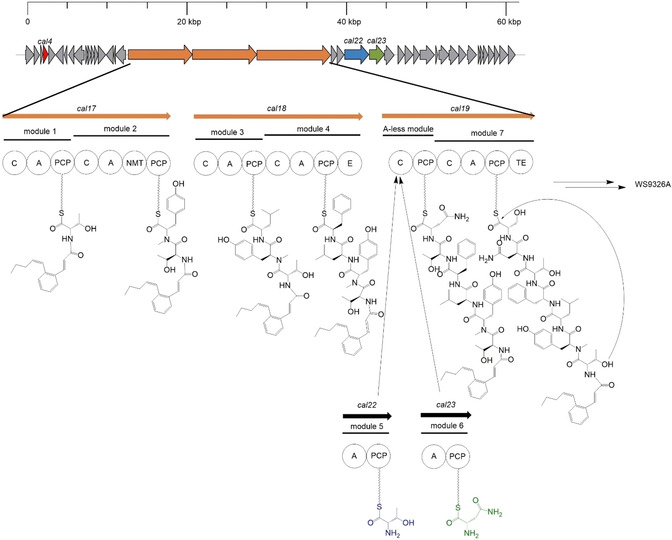
Biosynthetic gene cluster and proposed biosynthesis of WS9326A. *Cal4* is marked in red, *cal17*, *cal18*, *cal19* are marked in orange. *Cal22_Thr_* and threonine are marked in blue and *cal23_Asn_* and asparagine are marked in green.

Interestingly, the gene cluster encoding the NRPS deviates from the collinearity rule (Figure 2). A pair of genes, *cal22_Thr_* and *cal23_Asn_* encoding A‐PCP‐di‐domains is located downstream of the main NRPS genes and predicted to specify loading of threonine 5 (^5^Thr) and ^6^Asn of WS9326A.^21^ A *trans* working mechanism for these two A‐domains was proposed.^8^

In this study, we investigated the functions of the two *trans* A‐domains Cal22_Thr_ and Cal23_Asn_. We performed gene inactivation experiments to confirm the involvement of the *trans* A‐domains in WS9326A biosynthesis and performed *in vitro* enzyme activity assays to analyze their substrate specificities.

## Results and Discussion

### The *trans* A‐domains Cal22_Thr_ and Cal23_Asn_ are necessary for WS9326A biosynthesis in *Streptomyces calvus* ATCC 13382

**Figure 3:**
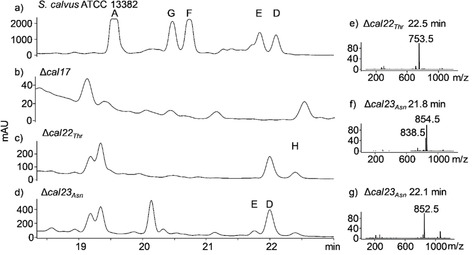
HPLC‐MS (ESI‐) analysis of culture extracts from *S. calvus* ATCC 13382 and mutant strains with UV‐visible absorbance detection at *λ*=279 nm. a) Extract from *S. calvus* ATCC 13382. Peaks corresponding to WS9326A (19.5 min), G (20.5 min), F (20.75 min), E (21.8 min) and D (22.1 min) are indicated. b) Extract from *S. calvus* Δ*cal17*. c) Extract from *S. calvus* Δ*cal22*. A peak corresponding to WS9326H (22.5 min) is indicated. d) Extract from *S. calvus* Δ*cal23*. Peaks corresponding to WS9326E (21.8 min) and WS9326D (22.1 min) are indicated. e) Mass spectrum (ESI‐) of Δ*cal22_Thr_* extract at *t*_R_=22.5 min. f) Mass spectrum (ESI‐) of Δ*cal23_Asn_* extract at *t*_R_=21.8 min. g) Mass spectrum (ESI‐) of Δ*cal23_Asn_* extract at *t*_R_=22.1 min.

The *S. calvus* WS9326A biosynthetic gene cluster^20^, ^21^ is essentially identical to that found in *S. asterosporus* DSM 41452.^8^ The *S. calvus* WS9326A gene cluster consists of 41 open reading frames spanning over 60.7 kb (Figure 2). In Table 1, all relevant genes for this study are listed (gene numbering from Johnston et al.^21^). The WS9326A cluster is divided into two parts. Genes required for the biosynthesis of the (Z)‐pentenylcinnamoyl moiety are located in the second part (*cal24*–*cal40*). In contrast, all genes responsible for NRP biosynthesis are located in the upstream part of the gene cluster, comprising the main NRPS genes *cal17*, *cal18* and *cal19*, spanning over one third of the gene cluster. Each main NRPS gene encodes two modules, resulting in six modules total (Figure 2). Module 1 is predicted to activate threonine according to NRPSpredictor2.^6^ Module 2 is predicted to specify tyrosine, whereas Module 3 is predicted to specify a hydrophobic amino acid, consistent with ^3^Leu of WS9326A. Module 4 is predicted to specify phenylalanine. An unusual A‐less module, consisting of a C‐PCP‐di‐domain, is encoded within *cal19*. This is followed by the last module, Module 7, that is predicted to specify serine.^6^, ^7^ In order to confirm the involvement of the gene cluster in WS9326A biosynthesis, *cal17* was disrupted by single crossover. *Cal17* is one of the three main NRPS genes, encoding the first and the second module, incorporating ^1^Thr and (*E*)‐2,3‐dehydrotyrosine (^2^ΔTyr), respectively. A mutant strain was obtained and its WS9326A production was analyzed by HPLC‐MS (ESI) in negative ion mode. Compared to the wildtype strain, which shows production of WS9326A and the congeners D, E, F and G (Figure 3a), the *cal17* mutation led to a complete loss of production of these compounds (Figure 3b). None of the detected peaks in the *cal17* mutant extract corresponded to masses for WS9326A or its congeners. This result confirms that WS9326A is encoded by the predicted gene cluster. Two more modules, specifying the adenylation of threonine and asparagine, are necessary for WS9326A biosynthesis. Two genes, *cal22_Thr_* and *cal23_Asn_*, are located outside the main NRPS genes. *Cal22_Thr_* and *cal23_Asn_* encode A‐PCP‐di‐domains, which are predicted by NRPSpredictor2 to adenylate threonine and asparagine, respectively. We performed knockout experiments of genes *cal22_Thr_* and *cal23_Asn_* to confirm their involvement in WS9326A biosynthesis. Gene disruption led to two mutant strains, *S. calvus* Δ*cal22_Thr_* and *S. calvus* Δ*cal23_Asn_*. After fermentation for seven days, the crude extract was analyzed for secondary metabolite production by HPLC‐MS (ESI‐).

**Table 1:** Relevant genes of *S. calvus* ATCC 13382 WS9326A gene cluster. Comparison with *S. asterosporus* DSM 41452 genes and their predicted functions.

*S. calvus*	*S. asterosporus*	Predicted
genes	genes	functions
*cal4*	*sas4*	MbtH‐like protein
*cal17*	*sas17*	Modules 1 and 2
*cal18*	*sas18*	Modules 3 and 4
*cal19*	*sas19*	A‐less module and Module 7
*cal22_Thr_*	*sas22*	Module 5
*cal23_Asn_*	*sas23*	Module 6

In neither mutant strain was WS9326A detected. Instead, *S. calvus* Δ*cal22_Thr_* produced a small amount of a compound with a retention time of 22.5 min and *m/z* 753.5 ([*M*–H]^−^; Figure 3c and e). This ion corresponds to a new linear congener WS9326H which relative to WS9326A is missing ^5^Thr, ^6^Asn, ^7^Ser and one degree of unsaturation. High‐resolution MS is consistent with the molecular formula for this congener and MS/MS fragmentation confirmed the presence of ^3^Leu and ^4^Phe (Figure S1 in the Supporting Information). We propose that tyrosine rather than (*E*)‐2,3‐dehydrotyrosine is present at position 2 in WS9326H as observed in WS9326B. The structure of WS9326H is consistent with premature hydrolysis of the peptide from module 4 in the absence of Cal22_Thr_ (module 5). *S. calvus* Δ*cal22_Thr_* also produces a compound with the same retention time as WS9326D (22 min, Figure 3c), but the observed molecular ion for this compound (*m/z* 265 [*M*–H]^−^) does not appear to be related to WS9326A or its congeners. In contrast, *S. calvus* Δ*cal23_Asn_* produces two compounds (Figure 3d) in small quantities with retention times and *m/z* values consistent with WS9326D (*m/z* 852.2 [*M*–H]^−^) and E (*m/z* 838.5 [*M*–H]^−^; Figure 3f and g). In the same chromatographic peak containing WS9326E, an ion of *m/z* 854.5 is also observed (Figure 3f), which matches to the mass of WS9326D plus 2 Da. This likewise may correspond to a WS9326D derivative where tyrosine rather than (*E*)‐2,3‐dehydrotyrosine occurs at position 2. These congeners are consistent with premature hydrolysis of the polypeptide chain from the A‐less module after incorporation of ^5^Thr by Cal22_Thr_.

Because the Δ*cal22_Thr_* and Δ*cal23_Asn_* mutants were derived from single cross‐over mutations, a polar effect might be expected on the expression of the fatty acid biosynthetic genes, all of which reside downstream of *cal22* and *cal23*.^8^ However, this does not appear to be the case as all of the congeners produced by the mutants contain the (Z)‐pentenylcinnamoyl side chain.

The structures of WS9326D, E, F and G found in *S. asterosporus* DSM 41452 (Figure 1) also support the hypothesis of the *trans* working A domains. Asparagine and serine are not part of WS9326D and WS9326E.^8^ After the incorporation of the first *trans* adenylated amino acid ^5^Thr, it appears that peptide elongation stops and the peptide is released without cyclization. Moreover, WS9326F and WS9326G contain ^6^Asn, the second *trans* adenylated amino acid, but the incorporation of the last amino acid, ^7^Ser, is not observed.^8^ Hence, these congeners were released without cyclization from NRPS machinery as well. We propose that the unusual NRPS arrangement might lead to premature arrest of NRP synthesis after the incorporation of the *trans* adenylated amino acids.

### Expression and purification of Cal22_Thr_ and Cal23_Asn_ with and without MbtH‐like proteins

These findings encouraged us to analyze the specificity of the two *trans* A‐domains Cal22_Thr_ and Cal23_Asn_ *in vitro*. Therefore, we purified both proteins and tested all proteinogenic amino acids as putative substrates via an enzymatic *in vitro* assay. We used the *E. coli* BL21(DE3) Δ*ybdZ* mutant strain for A‐domain production.^12^ Both Cal22_Thr_ and Cal23_Asn_ were expressed with N‐terminal His_6_‐tags. We tried to produce and purify both A‐domains without auxiliary MLPs and were unsuccessful in both cases. After purification with Ni‐NTA affinity chromatography and gel filtration, nearly no soluble Cal23_Asn_ was produced (Figure S2). In the purification of Cal22_Thr_ low yields of soluble protein were obtained (Figure S2). In both cases the majority of protein occurred as an insoluble inclusion body.

Next, we coexpressed each A domain with *cal4*, which is predicted to encode a MLP. We hypothesized that coproduction of Cal4 with Cal22_Thr_ and Cal23_Asn_ would be necessary to afford folded and functional A domains. Together with each A domain gene, *cal4* was cloned into the dual expression vector pETDuet1, without a His_6_‐tag‐specifying sequence. Purified yields of both soluble A domains improved significantly upon coexpression of Cal4. On average, purified Cal23_Asn_ yield was 3.75 mg/L culture (Figure S4). Notably, Cal4 copurified with Cal23_Asn_ as shown by SDS‐PAGE (Figure S4), indicating that Cal4 and Cal23_Asn_ form a strong complex. Similarly, the yield of soluble Cal22_Thr_ was also improved, yielding 1.01 mg/L culture of soluble protein. However, SDS‐PAGE analysis revealed that in this case Cal4 did not copurify with Cal22_Thr_, indicating that these proteins do not form a strong complex (Figure S3).

These results are consistent with the findings of Boll et al., who stated that MLPs are required for some A‐domains.^12^ Miller et al. elucidated the crystal structure of EntF together with the *E. coli* derived MLP YbdZ. They demonstrated that the MLP dependent A‐domain EntF interacts with two highly conserved YbdZ tryptophan residues, which form a pocket. To interact with MLPs, A‐domains require a conserved proline or alanine residue. An EntF alanine residue is compatible with the tryptophan pocket.^13^ In comparison, PA1221, an A‐PCP didomain from *Pseudomonas aeruginosa*, which is not dependent on MLPs, contains a glutamate residue at this position instead of alanine or proline.^27^ The authors hypothesized that a large polar residue prevents MLP binding to A domains.^13^ Cal4 contains both highly conserved tryptophan residues, Trp‐26 and Trp‐36, comprising the hydrophobic pocket (Sequence S1). Cal23_Asn_ has an alanine residue, Ala‐384, that is compatible with the tryptophan pocket of Cal4 (Figure S5). A third highly conserved tryptophan residue is also found in MLP's. It is present both in YbdZ and Cal4 (Trp‐56). It interacts with three amino acid residues from MLP dependent A‐domains, such as EntF. Proline, threonine and alanine are highly conserved in MLP dependent A‐domains, forming a PXXTA pocket, where X represents a variable amino acid.^13^ In contrast, no PXXTA pocket is found in the MLP independent PA1221.^27^ Cal23_Asn_ contains all three conserved amino acids (Pro‐375, Thr‐378, Ala‐379), that form the PXXTA pocket. Formation of a strong Cal23_Asn_ and Cal4 complex can be interpreted as a result of both proteins having all the conserved amino acid residues that comprise the interaction motif. Overall, the compatible sequence motifs, the increase in Cal23_Asn_ yield upon coexpression with Cal4 and formation of a complex between these two proteins, indicate that Cal23_Asn_ is dependent on MLPs for proper folding. However, as it was not possible to purify Cal23_Asn_ without Cal4, it was not possible to determine if the presence of Cal4 increases Cal23_Asn_ activity.

In contrast, sequence analysis suggests that Cal22_Thr_ is MLP independent. Cal22_Thr_ has a threonine residue, Thr‐746 (Figure S5), instead of the expected alanine that engages the tryptophan pocket of Cal4. Additionally, the PXXTA pocket contains a glutamate (Glu‐741), instead of alanine. According to Miller et al.’s hypothesis, Cal22_Thr_ would appear to be MLP independent, which would appear to be consistent with the absence of a strong interaction between Cal22_Thr_ and Cal4. Nevertheless, Cal22_Thr_ was only produced in high amounts as a soluble protein when coexpressed in *E. coli* with Cal4, indicating the requirement of an MLP during protein folding. We therefore hypothesize, that the MLP Cal4 does not strongly bind to Cal22_Thr_, but is instead involved and required for protein folding. The exact interaction mechanism of Cal22_Thr_ with Cal4 remains to be elucidated.

### Specificity of adenylating enzymes Cal22_Thr_ and Cal23_Asn_

**Figure 4:**
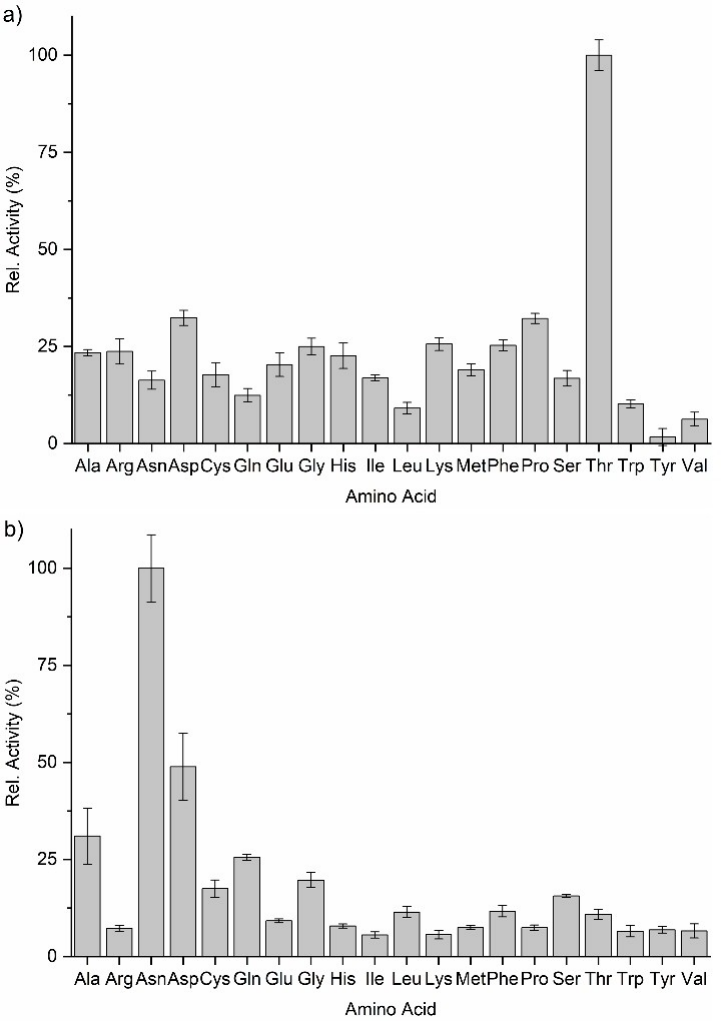
Amino acid selectivity of the *trans* A domains. a) Adenylation activity of Cal22_Thr_ after 3 h of reaction with 0.2 mM amino acids and 0.15 mM ATP. b) Adenylation activity of Cal23_Asn_/Cal4 complex after 3 h of reaction with 0.2 mM amino acids and 0.15 mM ATP. Activities are expressed relative to the most active amino acid substrate. Mean values of three independent experiments and standard deviations are shown.

Purified Cal22_Thr_ and the Cal23_Asn_/Cal4 complex were investigated by using an adenylation assay to evaluate their substrate specificities. In this assay, the reaction of ATP with an amino acid to form an amino acyl adenylate was measured by detecting the pyrophosphate (PP_i_) coproduct. Preliminary experiments were carried out to determine the optimal reaction conditions. Activity was measured over time (Figures S6 and S7). Moreover, different enzyme concentrations were tested, showing selectivity for their putative substrates at higher and lower enzyme concentrations for both A domains (Figures S8 and S9). All 20 proteinogenic amino acids were then tested as putative substrates. Cal22_Thr_ was observed to be most specific towards its predicted substrate threonine (Figure 4a). Likewise, the Cal23_Asn_/Cal4 complex was most active towards asparagine, the predicted substrate of the A‐domain (Figure 4b). Significant activity was also observed in both A‐domains towards other amino acids. However, no analogues of WS9326A or its congeners have been detected that have amino acids other than Thr and Asn at positions 5 and 6, respectively. This indicates that the *in vitro* assay does not fully capture the specificity of Cal22_Thr_ and Cal23_Asn_ in the context of the full NRPS system. Such promiscuous adenylating activity has been observed previously for A‐domains *in vitro*, including MLP dependent ones. This includes the Arg activating A domain from the nocardicin A pathway,^28^ the Val activating A‐PCP didomain (PA1221) from *P. aeruginosa*,^27^ and the Val activating HrmP2_A_ from the hormaomycin pathway.^29^ Remarkably, HrmP2_A_ appears to specify Ile rather than Val *in vivo*, as Val is not observed in the structure of hormaomycin. This indicates that in some cases the specificities of A‐domains towards amino acids can become more stringent or change significantly *in vivo*.

## Conclusion

In this study, we determined the roles of the A domains Cal22_Thr_ and Cal23_Asn_ in the biosynthesis of WS9326A. We confirmed the involvement of both *trans* A domains in WS9326A biosynthesis in *S. calvus* through gene disruption. The MbtH‐like protein Cal4 is shown to promote soluble expression of both A domains and Cal23_Asn_ is observed to form a stable complex with Cal4. Finally, we demonstrated in an *in vitro* assay that both A domains preferentially adenylate their predicted substrates, threonine and asparagine, which comprise residues 5 and 6 of WS9326A. Overall, this work demonstrates that Cal22_Thr_ and Cal23_Asn_ act in a *trans* fashion with the NRPS machinery to synthesize WS9326A.

## Experimental Section

**Strains, media, chemicals and bioinformatic analysis**: *Streptomyces calvus* ATCC 13382 was purchased from ATCC 13383. *E. coli* ET12567×pUZ8002 was used for conjugational transfer of plasmids into *Streptomyces. E. coli* BL21(DE3) Δ*ybdZ* was obtained from Boll et al.^12^ Media components were all purchased from Roth, except soy flour (Hensel Vollsoja) and BactoSoytone (BD). The following media were used lysogeny broth (LB; ready‐mix, pH 7.3), Terrific broth (TB; 12 g tryptone, 24 g yeast extract, 0.04 g glycerol, 12.54 g KH_2_PO_4_, 15 g K_2_HPO_4_, pH 7.0), Tryptic soy broth (TSB; ready‐mix), SG^+^ (20 g glucose, 10 g BactoSoytone, 2 g CaCO_3_, 1 mg CoCl_2_, 0.1 % (m/V) Valine, pH 7.2), MS (20 g d‐mannitol, 20 g soy flour, 10 mm MgCl_2_. The values were used to prepare 1 L medium. The pH was adjusted with HCl (1 m) or NaOH (1 m) before sterilization. For solid medium, agar‐agar (21 g) was added before sterilization. The final antibiotic concentrations were: 100 μg/mL ampicillin; 50 μg/mL apramycin; 200 μg/mL fosfomycin; 50 μg/mL kanamycin. [^33^P] ATP (1.11 TBq/mmol) was obtained by Hartmann Analytic. Gene cluster analysis was carried out using antiSMASH.^30^ Putative adenylation domain substrate specificities were predicted by NRPSpredictor2.^6^

**Generation of single crossover mutants** ***S. calvus*** **ATCC 13382 Δ*cal17*, Δ*cal22***_***Thr***_ **and Δ*cal23***_***Asn***_: Standard protocols for DNA isolation and manipulation procedures in *E. coli* and *Streptomyces* were applied. Primers were synthesized by Eurofins Co., Ltd. (Germany) and are listed in Table S1. Restriction enzymes and DNA ligase were acquired from NEB Biotechnology Co. Ltd. (England). Plasmid and PCR Product isolation kits were purchased from Promega Corporation (USA). All PCR products from *S. calvus* genomic DNA were amplified using Phusion polymerase. For construction of pKC1132‐*cal17*, primers *cal17*‐f and *cal17*‐r were used to amplify an internal fragment of *cal17* gene. The PCR product was isolated and digested with PstI and NotI. The respective restriction sites were located within the 2.8 kb *cal17* internal fragment. Restriction resulted in a 1.8 kb fragment. It was ligated into the corresponding restriction sites of pKC1132 vector, resulting in pKC1132‐*cal17*. To construct pKC1132‐*cal22*, the 1 kb internal fragment, obtained by PCR using the primers *cal22*‐f and *cal22*‐r, was ligated into SmaI digested pUC19. By restriction with KpnI and BamHI the fragment was isolated and ligated into pKC1132. To obtain pKC1132‐*cal23*, a *cal23* internal fragment was amplified by PCR using the primers *cal23*‐f and *cal23*‐r. After ligation into SmaI linearized pBluescriptSK(−), the fragment was excised using PstI and BamHI and cloned into linearized pKC1132, resulting in pKC1132‐*cal23*. All inactivation constructs were transformed into *E. coli* ET12567×pUZ8002 and transferred into *S. calvus* ATCC13382 by intergeneric conjugation following standard protocols. Apramycin resistant mutants (50 μg/mL) were isolated. After genomic DNA isolation, the obtained mutants were tested for target gene disruption by PCR. For confirmation of *cal17* disruption, primers Ins‐cal17‐f and lac‐r were used. Disruption of *cal22* was confirmed by PCR using the primers out‐cal22‐f and out‐cal22‐r. A PCR with the primers cal22‐f and out‐cal23‐r confirmed insertion of pKC1132‐*cal23* within the gene *cal23*.

**Construction of plasmids for protein production**: Both genes, *cal22* and *cal23*, consist of the adenylating enzyme together with the PCP‐domain. We amplified the complete ORF, including the PCP‐domain. To construct pET28a‐*cal22*, the gene was amplified by the primers ForAThr_BamHI and Rev‐AThr_EcoRI (Table S1). pET28a was restricted with the same enzymes and the digested PCR fragment was cloned into pET28a, resulting in pET28a‐*cal22*. pETDuet1‐*cal22+cal4* was constructed by linearization of pETDuet1 with NdeI and XhoI. The PCR fragment *cal4* was amplified with the primers For‐MbtH_NdeI and Rev‐MbtH_XhoI, restricted with the same enzymes and ligated into linearized pETDuet1, resulting in pETDuet1‐*cal4*. This plasmid was digested with BamHI and EcoRI. *cal22* was excised from pET28a‐*cal22* using BamHI and EcoRI and ligated into pETDuet1‐*cal4*. Additionally, one base pair was added in front of the start codon to avoid frameshift. The vector was linearized with NcoI and BamHI. A complementary oligomeric pair (catgggcagcagccatcaccatcatcaccacagcctagg and gatcctaggctgtggtgatgatggtgatggctgctgcc) replaced the excised part. The oligomeric pair anneals under a defined temperature program (30 min 37 °C, 5 min 95 °C, temperature is lowered in 5 °C steps and incubated for 1 min up to 25 °C). The resulting oligomeric pair is complementary to BamHI and NcoI restriction sites. The sample is diluted 1 : 200 and ligated into the linearized plasmid, resulting in pETDuet1‐*cal22*+*cal4*. The primers For‐AAsn_BamHI and Rev‐AAsn_NotI were used to amplify *cal23*. After gel purification, the fragment was digested with BamHI and NotI and cloned into linearized pETDuet1, resulting in pETDuet1‐*cal23*. This plasmid was restricted with NdeI and XhoI. *Cal4* was amplified with For‐MbtH_NdeI and Rev‐MbtH_XhoI and digested using the restriction sites introduced by the primers. Ligation into pETDuet1‐*cal23* resulted in pETDuet1‐*cal23*+*cal4*. All A‐domains were expressed as N‐terminally His_6_‐tagged fusion proteins, while *cal4* without a His_6_‐tag specifying sequence.

**Fermentation and extraction of WS9326A**: 30 mL TSB liquid medium was inoculated with a small loop of *Streptomyces* spores. The strain was cultivated until saturation at 28 °C and 180 rpm. 30 mL SG^+^ medium was inoculated with 1 mL preculture. This culture was grown at 28 °C and 180 rpm for 7 days and then centrifuged (10 min, 5003 g, 20 °C). After pH adjustment to 4, the supernatant was extracted with ethyl acetate (1 : 1) in a separation funnel for 20 min under constant shaking. The organic solvent was filtered into a round bottom flask and was evaporated under reduced pressure. The crude extract was solved in 500 μL Methanol and filtered through a PVDF membrane (0.45 μm). 50 μL were analyzed with an Agilent 1100LC series LC/MS system with electrospray ionization (ESI). Separation occurred on an XBridge C_18_ precolumn (20×4.6 mm, 3.5 μm particle size) and an XBridge C_18_ main column (100×4.6 mm, 3.5 μm particle size) maintained at 30 °C. Detection was carried out with a diode array detector at wavelengths 254/360, 480/800, 360/580, and 430/600 nm. The mobile phase comprised solvent A (H_2_O+0.5 % (*v*/*v*) acetic acid) and solvent B (acetonitrile and 0.5 % (*v*/*v*) acetic acid). For elution, a gradient method was used (4 min: 95 % solvent A+5 % solvent B; 5–20 min: 5–95 % solvent B; 20–22 min: 95 % solvent B; 23–28 min: 5 % solvent B; 0.5 mL/min). MSD settings were as follows: Acquisition‐mass range: 150–1000 m/z, MS scan rate: 1/s, MS/MS scan rate: 2/s, fixed collision energy: 20 eV, ion source drying gas temperature: 350 °C, drying gas flow: 10 L/min, nebulizer pressure: 35 psig, ion source mode: API‐ES, capillary voltage: 3000, MS detector autotuning: Agilent tuning solution is positive and negative mode before measurement. LC (DAD) and MS data were analyzed with ChemStation Rev. B. 09.03 software (Agilent). MS/MS measurements were performed with an Agilent 1290 Infinity II LC and 6045 LC/QTOF system. Separation occurred on a Zorbax Eclipse Plus C18 RRHD 2.1×50 mm, 1.8 micron column by Agilent. The mobile phase comprised solvent C (H_2_O+0.1 % (*v*/*v*) formic acid) and solvent D (acetonitrile and 0.1 % (*v*/*v*) formic acid). For elution, a gradient method was used (0–14 min: 90–0 % solvent C and 10–100 % solvent D; 14–19 min: 100 % solvent D; 19–20 min: 90 % C; 20–23 min: 90 % solvent C).

**Protein purification**: Due to its lack of the *E. coli* derived MbtH‐like protein YbdZ, the host strain *E. coli* BL21(DE3)Δ*ybdZ* was used for protein production, kindly provided by Dr. Bertold Gust, University of Tübingen. 1 L TB medium (supplemented with apramycin and ampicillin for pETDuet1 vectors and apramycin and kanamycin for pET28a vector) was inoculated with 10 mL of an LB overnight culture. The culture was incubated at 37 °C until OD_600nm_ reached 0.4–0.6. Protein production was induced with 0.5 mM Isopropyl‐β‐D‐thiogalactopyranoside (IPTG) for Cal22_Thr_ production and 1 mM IPTG for Cal23_Asn_ production, respectively. The cultures were grown for 16 h at 20 °C and then harvested by centrifugation (8000 rpm, 11325 g, 20 min, 4 °C). The cell pellet was resuspended in lysis buffer in a 3 : 1 ratio (Cal22_Thr_: 50 mM Tris, 150 mM KCl, 10 % (*v*/*v*) glycerol, 10 mM imidazole, 5 mM β‐mercaptoethanol 1 mM phenylmethylsulfonyl fluoride (PMSF), pH 7.5; Cal23_Asn_: 50 mM Tris, 300 mM NaCl, 10 mM imidazole, 1 mM PMSF, pH 7.5). Cells were lysed by French Pressure Cell Press. Cell debris was pelleted by centrifugation (10 000 rpm, 17969 g, 20 min, 4 °C). The supernatant was incubated with 1 mL Ni‐NTA agarose beads (Roth) over 30 min at 4 °C. The sample was washed with buffer containing 20 mM imidazole. Finally, His‐tagged proteins were eluted with 250 mM imidazole containing buffer. Fractions were analyzed by SDS‐PAGE. Protein containing fractions were pooled and concentrated. Protein absorbance was measured with NanoDrop and concentration was calculated via extinction coefficient. Further purification was carried out by size exclusion chromatography, using a HiLoad® 16/600 Superdex® 200 prep grade with size exclusion buffers (Cal22_Thr_: 50 mM HEPES, 150 mM KCl, 10 % (*v*/*v*) glycerol, 1 mM DTT, pH 7.5; Cal23_Asn_: 25 mM Tris, 150 mM NaCl, 5 mM MgCl_2_). The protein containing fractions were concentrated using a Vivaspin 20 concentration unit and flash‐frozen in liquid nitrogen, stored at −80 °C and used only once after thawing.

**Adenylation assay**: The adenylation assay was carried out to determine the specificity of the A‐domains of Cal22_Thr_ and the Cal23_Asn_/Cal4 complex. Each reaction consisted of 30 μM of the purified protein in buffer (50 mM Tris, 150 mM NaCl, 1 mM MgCl_2_) and 0.2 mM amino acid. The reaction was initiated by the addition of [^33^P] ATP to a final concentration of 0.15 mM. The total volume of the reaction was 20 μL. The reaction was incubated at 25 °C for 3 h then stopped by addition of 800 μL of a slurry of activated charcoal (5 % *w*/*v*) in 20 mM H_3_PO_4_. After 10 min incubation on ice, the charcoal was removed by centrifugation (13 500 rpm, 2.3×1000 g, 10 min, 4 °C) and 200 μL of the [^33^P] PP_i_ containing supernatant was transferred to 3 mL scintillation liquid (Rotiszint® eco plus). Radioactivity was measured in a scintillation counter. A calibration curve was measured to determine the PP_i_ concentration. Preliminary experiments were carried out to determine the optimal assay conditions with respect to time and enzyme concentration. Measured values were multiplied by 4, due to the transfer of a quarter of the supernatant in each scintillation liquid vial. No amino acid was added for blank measurements and the obtained blank values were subtracted from all other values. Radioactivity values were normalized relative to the amino acid that afforded the highest activity.

## Conflict of interest

The authors declare no conflict of interest.

## Supporting information

As a service to our authors and readers, this journal provides supporting information supplied by the authors. Such materials are peer reviewed and may be re‐organized for online delivery, but are not copy‐edited or typeset. Technical support issues arising from supporting information (other than missing files) should be addressed to the authors.

Supplementary
