# Supplementary material for: Role of Two Exceptional trans Adenylation Domains and MbtH‐like Proteins in the Biosynthesis of the Nonribosomal Peptide WS9324A from Streptomyces calvus ATCC 13382
Source: Chembiochem. 2020 Jun 18;21(18):2659–66. doi: 10.1002/cbic.202000142 (PMC7539972; doi:10.1002/cbic.202000142)
Supplement: Supplementary file 1 — Supplementary [file CBIC-21-2659-s001.pdf]

ChemBioChem

Supporting Information

Role of Two Exceptional *trans* Adenylation Domains and MbtH-like Proteins in the Biosynthesis of the Nonribosomal Peptide WS9324A from *Streptomyces calvus* ATCC 13382

Mirjam Bernhardt, Stefanie Berman, David Zechel, and Andreas Bechthold*

Supporting Information

Table of contents

Table S1. Primers used in this study………………………………………………………….. 2
Table S2. Plasmid information………………………………………………………………… 3
Table S3. Strains constructed and used in this study…………………………………………. 3
Figure S1……………………………………………………………………………………… 4
Figure S2……………………………………………………………………………………… 5
Figure S3……………………………………………………………………………………… 5
Figure S4……………………………………………………………………………………… 6
Figure S5……………………………………………………………………………………… 6
Figure S6……………………………………………………………………………………… 7
Figure S7……………………………………………………………………………………… 7
Figure S8……………………………………………………………………………………… 8
Figure S9……………………………………………………………………………………… 8
Sequence S1. Cal4 amino acid sequence……………….……………………………………….. 9
Sequence S2. $Cal22_{Thr}$ amino acid sequence…………………………………………………..… 9
Sequence S3. $Cal23_{Asn}$ amino acid sequence…….……………………………………………… 9
References ……………………………………………………………………………………… 9

Table S1. Primers used in this study

Name	Sequence (5' → 3')
Primer for single crossover plasmids	
cal17-f	ATGAACGGGCTGACGTCGAACTC
cal17-r	CGACGACTTCTCCCTGATCGTGATG
cal22-f	TCGGCGTGTGCCTGGAACGCTC
cal22-r	GGAAGCCGCGCAGCTTGACCTGG
cal23-f	GGCGTGCTCAAGACCGGTGC
cal23-r	GCCGCGGATCTTGACCTGGTCG
Primer for PCR verification of gene disruption in *S. calvus* genomic DNA	
Ins-cal17-f	TGGAGACCGACCAGGTCGACAAGTG
lac-r	GCGATTAAGTTGGGTAACG
out-cal22-f	CCGGCTGACGTACGGCGAACTC
out-cal22-r	CAGGCCGGTGTCCTCGACGATG
cal22-f	TCGGCGTGTGCCTGGAACGCTC
out-cal23-r	GACCACGTAGCCGGCCAGGTAG
Primer for protein production plasmids	
For-MbtH_NdeI	GCGCATATGAGCACCAATCCTTTCGAGGAC
Rev-MbtH_XhoI	ATCTCGAGCTACTTGGCGCCTTCCATCTGG
For-AThr_BamHI	GTCCGGATCCGTGTCGATCACTCACCAGGAC
Rev-AThr_EcoRI	GTATGAATTCTCATCCGGCGCGGTCCG
For-AAsn_BamHI	AATGGATCCCCTGGTCCCGCGTGGTTCCATGCACGTGAACACAACCCAC
Rev-AAsn_NotI	AATATTGCGGCCGCTCACGACGCCGTCCCTCGGTC

Table S2. Plasmid information

Name	Description	Reference
pBluescriptSK(-)	Cloning vector for *E. coli*, *lacZ*(α-complementation), Amp^r	Stratagene
pUC19	Cloning vector for *E. coli*, Amp^r	[1]
pKC1132	Conjugation vector, non-replicative in *Streptomyces*, $Apra^r$	[2]
pKC1132-*cal17*	Single crossover plasmid	This study
pKC1132-*cal22*	Single crossover plasmid	This study
pKC1132-*cal23*	Single crossover plasmid	This study
pETDuet1	Dual protein production vector, Amp^r	Novagen
pETDuet1-*cal22+cal4*	Co-production of $Cal22_{Thr}$ and Cal4	This study
pETDuet1-*cal23+cal4*	Co-production of $Cal23_{Asn}$ and Cal4	This study
pUZ8002	Helper plasmid for conjugating plasmid containing the *oriT* sequence, $Kana^r$	[3]
pETDuet1-*cal23*	Heterologous production of $Cal23_{Asn}$	This study
pET28a-*cal22*	Heterologous production of $Cal22_{Thr}$	This study

Table S3. Strains constructed and used in this study

Name	Description	Reference
E. coli ET12567 x pUZ8002	Methylation-deficient *E. coli* strain for conjugation with the helper plasmid	invitrogen
E. coli BL21(DE3) Δ*ybdz*	YbdZ deficient *E. coli* strain for protein production	[4]
E. coli XL1-Blue	General cloning host	Stratagene
S. calvus ATCC 13382	Wild type strain of WS9326A producer	ATCC (no. 13382)
S. calvus ATCC13328 Δ*cal17*	*cal17* inactivation in the WT strain, $Apra^r$	This study
S. calvus ATCC13328 Δ*cal22*	$cal22_{Thr}$ inactivation in the WT strain, $Apra^r$	This study
S. calvus ATCC13328 Δ*cal23*	$cal23_{Asn}$ inactivation in the WT strain, $Apra^r$	This study

Figure S1. MS/MS analysis of Δ*cal22*$_{Thr}$ mutant. Peak corresponding to WS9326H (Figure 3c) is measured in positive ion mode (*m/z* 755.4). Proposed WS9326H structure comprises amino acids 1 to 4, missing [5]Thr, [6]Asn and [7]Ser. MS/MS spectrum after fragmentation with 5 eV in positive ion mode.
m/z value of 590.32 may correspond to a fragment missing [4]D-Phe. *m/z* value of 477.2 may correspond to a fragment also missing [3]Leu.

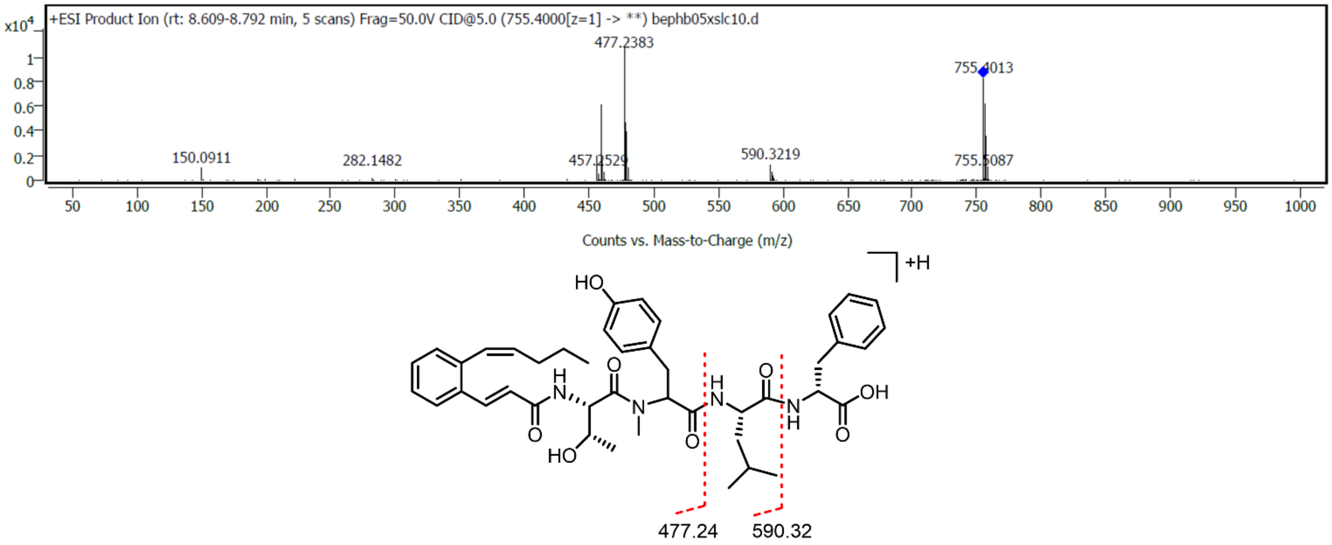

m/z = 755.4014 [M+H]+

Figure S2. a) SDS-PAGE of the Ni-NTA purified $Cal22_{Thr}$ (106 kDa). Protein ladder from Abcam (5 - 245 kDa) b) SDS-PAGE of Ni-NTA purified $Cal23_{Asn}$ (65 kDa) with and without Cal4. Protein ladder from Abcam (5 - 245 kDa); Lane 1 displays $Cal23_{Asn}$ purification together with Cal4 (8.2 kDa); Lane 2 displays $Cal23_{Asn}$ purification without Cal4

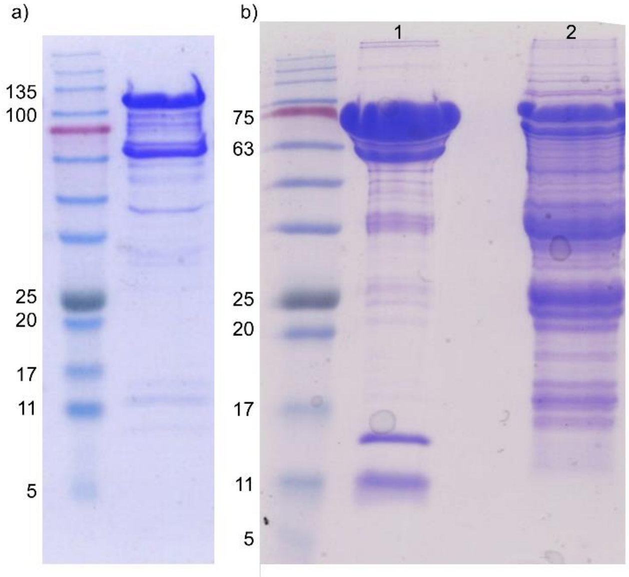

Figure S3. a) SDS-PAGE of Ni-NTA affinity chromatography elution fractions of $Cal22_{Thr}$ (106 kDa) after co-expression with Cal4 (8.2 kDa). Protein ladder from Abcam (5 - 245 kDa). b) Gel filtration chromatogram of co-expressed $Cal22_{Thr}$ with Cal4. The protein elutes between 60.5 mL to 74.0 mL. c) SDS-PAGE of gel purification fractions. Fraction 1 displays peak eluting at 55 mL, Fraction 2 - 4 display elution peak of gel purified $Cal22_{Thr}$

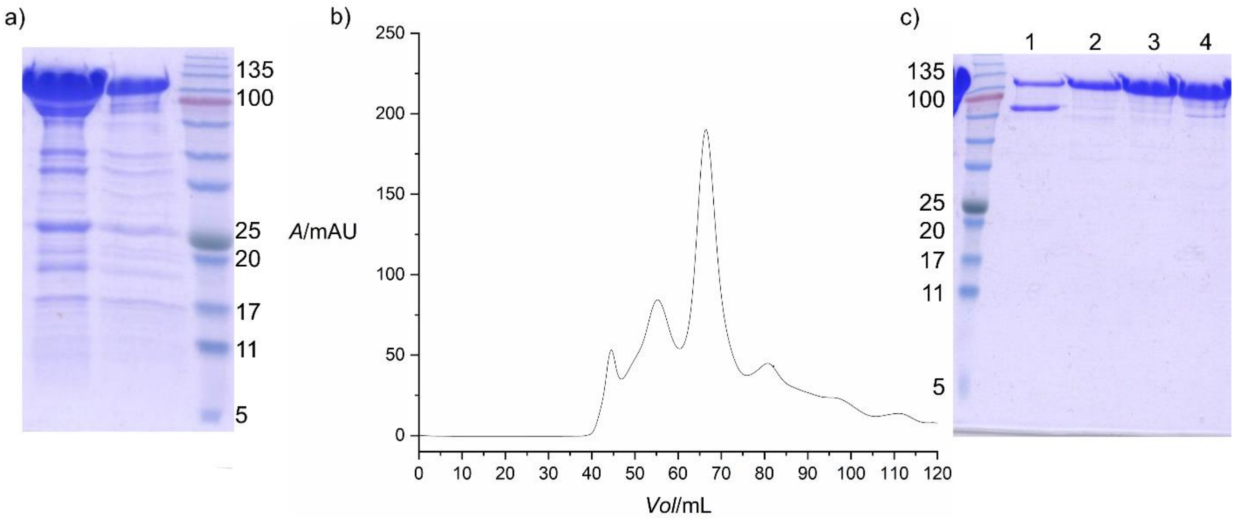

Figure S4. a) SDS-PAGE of Ni-NTA purified Cal23$_{Asn}$ (65 kDa) and Cal4 (8.2 kDa) after co-expression. Protein ladder from Abcam (5 - 245 kDa). b) Gel filtration chromatogram of Cal23$_{Asn}$ and Cal4. Protein elutes between 66.0 mL to 82.0 mL. c) SDS-PAGE of the gel purified Cal23$_{Asn}$ and Cal4

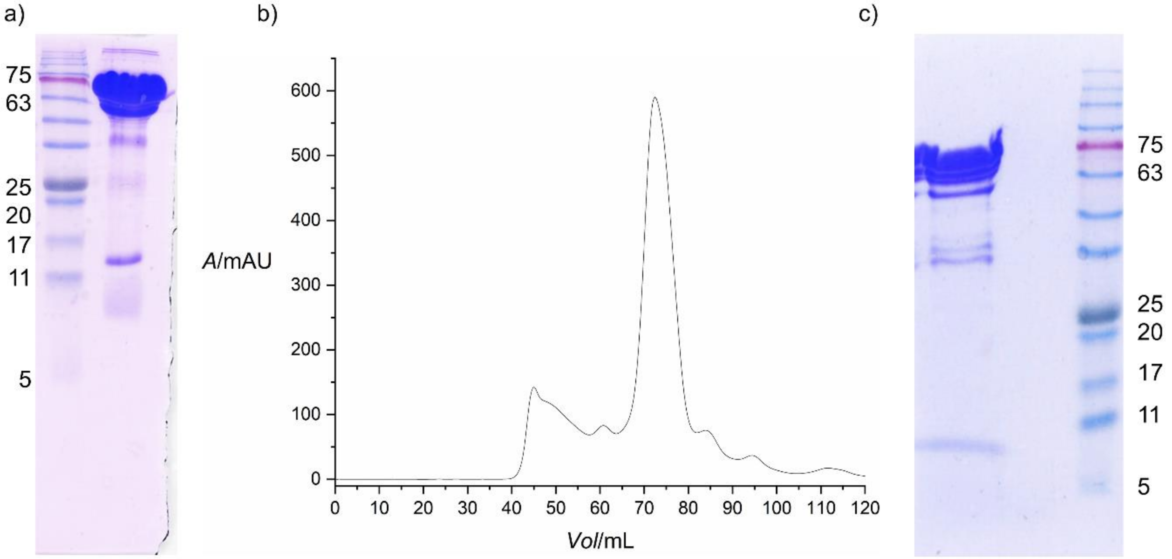

Figure S5. Sequence alignment of Cal22$_{Thr}$ and Cal23$_{Asn}$. The PXXTA pocket is highlighted. The conserved alanine residue of MLP dependent A-domains is highlighted. Cal23$_{Asn}$ comprises all conserved motifs. In comparison, Cal22$_{Thr}$ contains a glutamate instead of alanine in PXXTA pocket and threonine instead of the conserved alanine

```
Cal23      AGTEGELYIAGGQVARGYLGRPGLTAERFVAA-
Cal22      AGVAGEIYVGGAGVAAHYLNKPELTEQRFLTDV
           **. **:*:.*. **  **.:* ** :**::
```

Figure S6. Amino acid selectivity of $Cal22_{Thr}$ measured over time with 0.2 mM amino acids and 0.15 mM ATP. For Blank measurements, no amino acid was added. Obtained blank values were substracted from all other values. Activities are expressed relative to the most active amino acid substrate at maximum time.

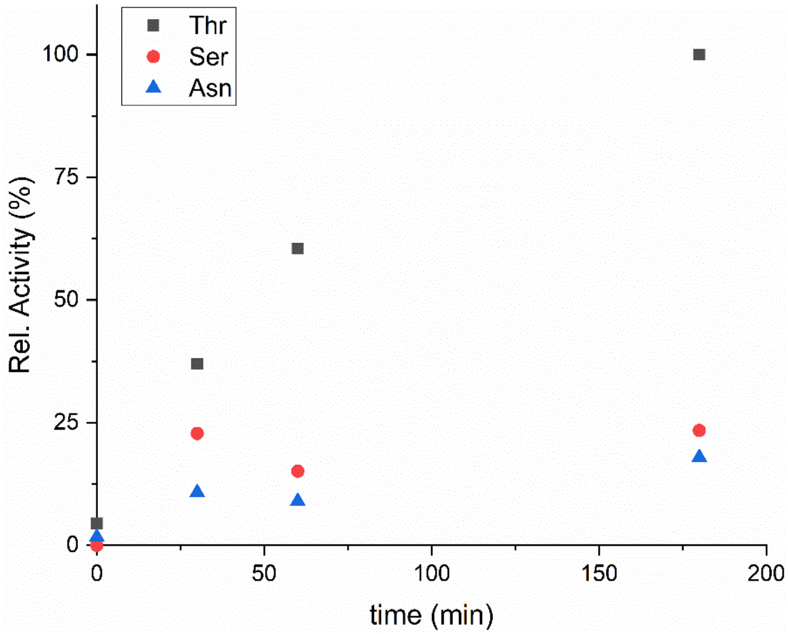

Figure S7. Amino acid selectivity of $Cal23_{Asn}$ measured over time with 0.2 mM amino acids and 0.15 mM ATP. For Blank measurements, no amino acid was added. Obtained blank values were substracted from all other values. Activities are expressed relative to the most active amino acid substrate at maximum time.

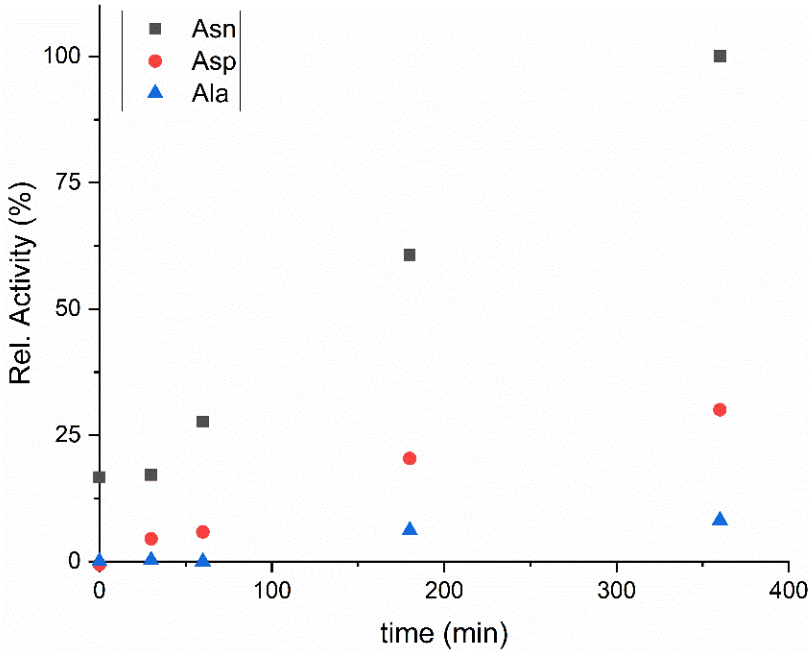

Figure S8. Amino acid selectivity of $Cal22_{Thr}$ with different enzyme concentrations. 0.2 mM amino acids and 0.15 mM ATP were used. For Blank measurements, no amino acid was added. Obtained blank values were substracted from all other values. Activities are expressed relative to the most active amino acid substrate at highest enzyme concentration.

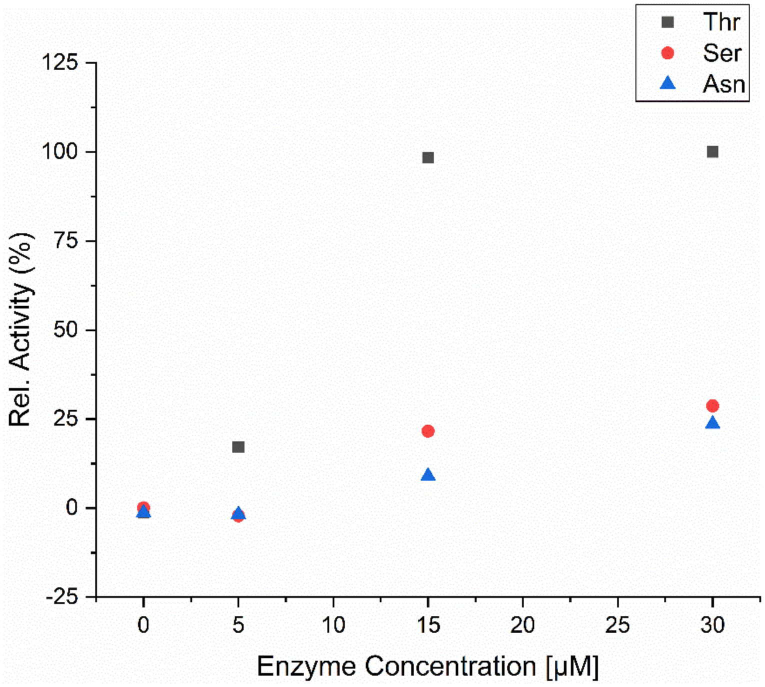

Figure S9. Amino acid selectivity of $Cal23_{Asn}$ with different enzyme concentrations. 0.2 mM amino acids and 0.15 mM ATP were used. For Blank measurements, no amino acid was added. Obtained blank values were substracted from all other values. Activities are expressed relative to the most active amino acid substrate at highest enzyme concentration.

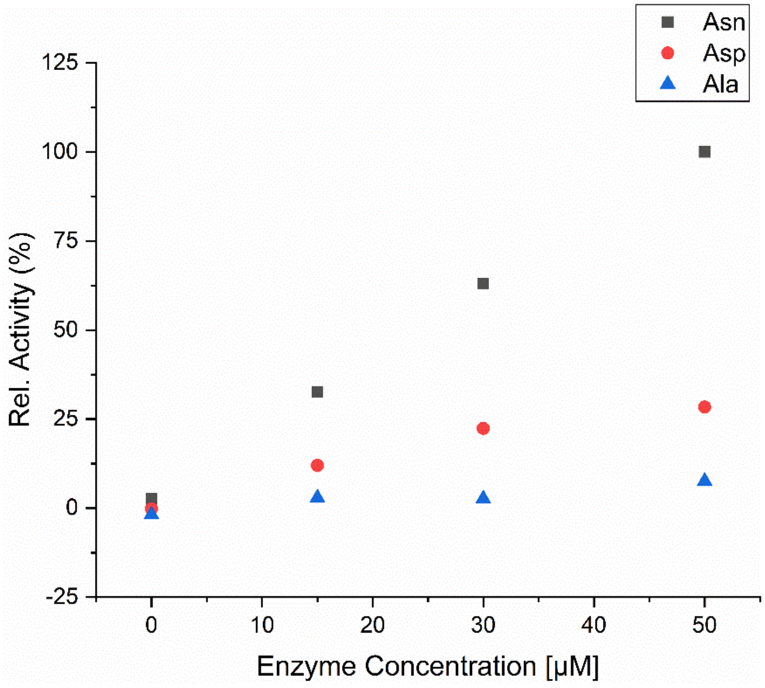

Sequence S1. Cal4 amino acid sequence. Conserved Trp-26, Trp-36 and Trp-56 are highlighted in grey

MSTNPFEDNDAQYRVLVNDEGQHSLWPAFAEVPAGWTVAHPEDSRQACLDYVNENWTDMRPLSLVRQ
MEGAK

Sequence S2. Cal22$_{Thr}$ amino acid sequence. Amino acids involved in PXXTA pocket are highlighted in blue. Amino acid bound to MLP tryptophan pocket is highlighted in red. All relevant amino acids are also marked in Figure S4

VSITHQDAAAARTPERAAAPAGAACHALRVRLTAGAAADGPDAVAARLTALTAGTGPSAGFPARLWTER
VPCGADAPAARRWRDRELGRPLPVHRDAPVRAVLLQYADGATDLVVVADRAALDTTALHAFTARLLGAE
QDTALTARTAATSPPAEPGPGGPVEQPDWGLGRPHGPDTPVTRVLPLPAGHPETDAESWATALGVVLA
RYEGVTRPALALLAPGTGAPGRPVVPAPVAADGGSTLGTVDKELRAALAAGPDGTATTAAVGLVLADRD
LSAGLPGADHAEYRPFQQPPFPVTVTVTRDATGGRALRCDHSARTLSDAVADQLLRHVSQVHRTLAEAP
DTLVADARPWDEEEAARLAAHRRSAPLPPFTPRRIDTAVAEIAAARPDAVAVRCEDTRLTYGELDRRADR
LAHALRALGVGDRDRVGVCLERSAEMIVVLLAVLKAGATYVPMDPAYPEDRLAYTTSDAGLTTVLTDRAD
FPGGTDTRVLTPAALEDLAPERCDGPPPSAAGADDPAYVIYTSGSTGRPKGCVVPHVNVLSLMAATTGD
FGLGADDTWTLFHSSAFDFSVWEIWGCLLTGGRLVVVPYWVSRSPEDFHRLLREERVTVLSQTPSAFAH
LVEVERDGADPAPVRLVVFGGEGLDPRMLLPWFDRHPETACRVVNMYGITETTVHVTAQTVTRAEALTA
SKSVGHALPGWWVRVADPDGRPLPAGVAGEIYVGGAGVAAHYLNKPELTEQRFLTDVRTGERLYRSGD
KGRLLPDGRLEHLGRLDSQVKLRGFRIELDEIRSVLLDDPQVTAAAVVLNRTDPDDAATAQLDAYVVMDG
DDTASVRQRATRFLPEYMVPSTVTALERMPLTTNGKLDTRRLPRPAAAPAPAAPAAPAATPAAAQAESA
AAQADTFEQVLLEVWREVLGVPVGPDDNFFDLGGNSLLAMRVGAALRRRGGHTVAMRDLYLRPTIRQLA
ADRAG

Sequence S3. Cal23$_{Asn}$ amino acid sequence. Amino acids involved in PXXTA pocket are highlighted in blue. Amino acid bound to MLP tryptophan pocket is highlighted in red. All relevant amino acids are also marked in Figure S4

MHVNTTHTTDDTMADPTLAELFQAQVARTPHAVAVHSGGQELDYAGLNRRANLLAHRLLGRGIGPEDVV
GVRLPRSADLLVALLGVLKTGAAYVPVDADCPAGRLAYIVEDTGLELMLVPDGEDADGTGEGPRTLSVPA
EVAAAEAAGGPAHDPTDADRPAPLTARNLAYVIYTSGSTGRPKGVTVQHDTLLRYLDFACDAYPGLARS
ALLHSPVAFDLTVTALYGPLLRGGHVRVGSLDREPEPGTPAGRPAFVKATPSHLPLLTVLPSSLSPTGELV
VGGEMLIGDVVERWRKTHPGATVVNEYGPTEATVGCCVFAIGPDDPVELGATPIGRPTPGTELHVLDERL
NPVPAGTEGELYIAGGQVARGYLGRPGLTAERFVAAPGGGRMYRTGDIARMRPDGLLEYLGRTDDQVKI
RGYRIELGEVTSVLAAQAGVRQAAVIAREDRSGDRYLAGYVVPEDGAAPDPAALREAVARHLPPYMVPS
AVVVVGELPLTSNGKLDKDALPEPPADDAPTGAAPVTEAETRLCALFAEVLGLPVVHLDDDFFDRGGDSL
RAAKLANKARKSGWTFGLRDVLELRTPRALAHAPGAPTEGAAS

[1] C. Yanisch-Perron, J. Vieira, J. Messing, *Gene* **1985**, *33*, 103–119.
[2] M. Bierman, R. Logan, K. O'Brien, E. T. Seno, R. Nagaraja Rao, B. E. Schoner, *Gene* **1992**, *116*, 43–49.
[3] T. Kieser, D. A. Hopwood in *Methods in Enzymology : Bacterial Genetic Systems*, Academic Press, **1991**.
[4] B. Boll, T. Taubitz, L. Heide, *The Journal of biological chemistry* **2011**, *286*, 36281–36290.
